# Views and experiences of the female condom in Australia: An exploratory cross-sectional survey of cisgender women

**DOI:** 10.1371/journal.pone.0246664

**Published:** 2021-02-19

**Authors:** Sarah E. Fenwick, Jessica R. Botfield, Prudence Kidman, Kevin McGeechan, Deborah Bateson

**Affiliations:** 1 Family Planning NSW, Ashfield, New South Wales, Australia; 2 Faculty of Medicine and Health, Sydney School of Public Health, The University of Sydney, Sydney, New South Wales, Australia; University of New South Wales, AUSTRALIA

## Abstract

**Background:**

The female condom is the only female-initiated form of protection against unintended pregnancy and sexually transmissible infections (STIs). However, use of this method in Australia is low. To better understand women’s views and experiences of the female condom, we conducted an interventional cross-sectional study.

**Methods:**

Cisgender women ≥16 years, heterosexually active and living in New South Wales were recruited through social media advertisements and email invitations to clients of a family planning service. Eligible participants were provided with three female condoms and invited to complete a follow-up survey. Survey responses for women who attempted to use at least one female condom were summarised using counts and proportions.

**Results:**

We recruited 556 women; few (30/556) had used the female condom before the study. There were 284 women who used, or attempted to use, a female condom during the study and completed the follow-up survey. Fifty-one percent (104/205) reported experiencing some difficulty in insertion, although only 46% (130/284) had seen an instructional demonstration. Approximately half (105/204) of women rated the sensation and comfort of the female condom as the same or better than the male condom, and 66% (137/204) reported that it provided the same or better lubrication. Approximately half of women said they would consider using the female condom again for STI prevention (51% (133/260)) or contraception (40% (103/260)), or would recommend to others (43% (112/260)).

**Conclusion:**

Findings highlight the need for increased health promotion and education regarding use of the female condom. To increase access it will be important to address method cost and availability in Australia. Future research should explore other perspectives of this method, including among the LGBTIQ+ community.

## Introduction

In Australia, approximately two-thirds of women of reproductive age use a method of contraception [1], most commonly the oral contraceptive pill and the male condom [1,2]. An alternative barrier method of contraception, the female condom (also called the internal condom), has been available in Australia since early 2000 [3,4], however, use of this method remains low [5]. The female condom is the only female-initiated method of protection for both unintended pregnancy and sexually transmissible infections (STIs). (Note: The terms ‘male condom’ and ‘female condom’ are used in this paper. While we recognise their limitations, these terms are commonly used in Australia and therefore used in the study presented).

The second-generation female condom (FC2) is the most widely distributed female condom on the market. It is a thin nitrile pouch which is inserted into the vagina before sex, preventing the transmission of sexual fluids between partners during sexual activity. In Australia, female condoms can be purchased from family planning clinics, sexual health clinics and online. Women’s health clinics and some pharmacies may also stock them. Although it is less accessible than the male condom as it is not widely available for purchase and is more expensive [6,7], it does offer several advantages. For instance, it can be used by those allergic or sensitive to latex [8], can be inserted into the vagina before sex and used with any type of lubricant [6], may enhance pleasure from clitoral stimulation [9,10], and it does not require an erect penis or immediate withdrawal after ejaculation [6,11]. While it has a higher failure rate than other modern methods of contraception, it is comparable to the male condom in terms of both contraceptive effectiveness [12,13] and STI prevention [6] and remains the only female-initiated method of dual protection [12]. Moreover, the female condom may allow women greater sexual autonomy to exercise control over their own contraception and prevention of STIs [14–16].

Female condom acceptability studies have been mainly conducted in low- and middle-income countries [17–20] or with marginalised populations [21–23]. To our knowledge, no female condom acceptability studies have been conducted in Australia. We therefore undertook a study in New South Wales (NSW), Australia, to gain a better understanding of the views and experiences of cisgender women regarding the female condom.

## Methods

An interventional cross-sectional study was undertaken between February and September 2019, to better understand women’s views and experiences of the female condom. As this was a small exploratory study, we limited the eligibility criteria to ‘females who are heterosexually active’. We recognise this was very targeted and excluded particular people, including men, trans and gender diverse people, and others in the LGBTIQ+ community.

Women were recruited through online social media advertisements (i.e., Facebook and Instagram) and email invitations to clients of a family planning service in NSW who had consented to be contacted for research purposes. Prospective participants first completed a short online survey to determine whether they met the following eligibility criteria: female, ≥ 16 years, living in NSW, and likely to engage in vaginal sex during the course of the study. While the female/internal condom can be used for anal sex to prevent STI transmission, this study focussed on those engaging in penis in vagina sex. We recognise that penis in vagina sex is engaged in by gender diverse people with a vagina who do not identify as female.

Eligible and consenting women were then provided with a study pack via post or collection from a participating family planning service. In addition to three female condoms (FC2), the study pack included optional resources including a factsheet and a link to a website where an animated instructional video and a step-by-step pictorial guide was hosted. Viewing these was not mandatory. Participants were also recommended to continue with their current method of contraception when participating in the study, unless male condoms were being used.

An email containing a link to the online survey was sent to participants several weeks after providing the study pack, as well as a maximum of three additional email or text message reminders. We included in the analysis only those who had used or attempted to use at least one of the provided female condoms. We defined attempted use as opening the female condom packet, or attempting insertion, but not proceeding to use it during sex. Those with attempted use were included as they would have direct experience of the female condom.

Data were collected across several measures including participant characteristics, awareness and knowledge, as well as experience and views of using the female condom during the study. Most questions used a multiple-choice design or rating system, and no questions were mandatory so there are some missing responses. Rating questions were across a 5-point scale where 1 indicated ‘very difficult’ or ‘a lot worse’ and 5 represented ‘very easy’ or ‘a lot better’. A rating of 3 was considered the neutral mid-point. Data were tabulated by frequency and expressed as a percentage using IBM SPSS Statistics for Windows 10, version 19.0. Ethics approval for the study was obtained from the Family Planning NSW Human Research Ethics Committee (approval #R2018-08). Consent was indicated by participants reading the online information sheet and proceeding to complete the survey; the ethics committee waived the need for parental consent for participants 16–18 years. No identifying information was asked in the survey.

## Results

### Participant characteristics

Five hundred and fifty-six women agreed to participate in the study and received the pack of female condoms. The majority (78%) were aged 35 or under (16–25 (n = 210); 26–35 (n = 221)) with the remaining aged 36–45 (n = 99), 46–55 (n = 21) and 56 or older (n = 5).

There were 284 women who completed the survey and, at a minimum, opened the female condom packet (51%; *n* = 284/556). Of these, most were born in Australia (90%), identified as heterosexual (67%) and as being in a committed relationship (83%). See Table 1 for survey participant characteristics.

**Table 1:** Participant characteristics (*n* = 284).

	*n*	%
**Age (years)**		
16–25	94	33
26–35	111	39
36–45	64	23
46–55	11	4
56–65	4	1
**Country of birth**		
Australia	255	90
Other	29	10
**Aboriginal/Torres Strait Islander background**		
No	271	95
Aboriginal	13	5
**Area of remoteness**^^^		
Major city	188	66
Inner regional	56	20
Outer regional/remote/very remote	40	14
**Sexuality**		
Heterosexual or straight	190	67
Bisexual	57	20
Queer	18	6
Other	9	3
Not sure	7	3
Lesbian, gay or homosexual	3	1
**Relationship status**		
Married/in a relationship	235	83
Single	33	12
Other	16	6
**Duration of relationship for those married/in a relationship**		
< 1 year	47	20
1–2 years	47	20
3–5 years	47	20
6–10 years	38	16
11–20 years	44	19
> 20 years	12	5

^ Participant postcodes were categorised by remoteness [following 24].

Approximately 9% (26/284) of women reported using the female condom before the study, where 7% (20/284) reported using the female condom “once or twice” before and 2% (6/284) reported using it “many times” before. Twenty-six percent (93/284) did not answer the question. The three most common forms of contraception participants had tried in the past were male condoms (95% (270/284)), the oral contraceptive pill (79% (223/284)) and withdrawal (49% (139/284)). These were also the main methods currently used (male condoms (58% (165/284)); oral contraceptive pill (22% (61/284)); withdrawal method (24% (68/284)) (see Table 2).

**Table 2:** Participant’s reported current use of contraception (*n* = 284).

Contraceptive method	*n*	%
Male condom	165	58
Oral contraceptive pill	61	22
Withdrawal	68	24
Implant/rod	20	7
IUD: copper or hormonal (Mirena)	34	12
Fertility awareness methods	20	7
Injection/Depo	4	1
Vaginal ring (Nuvaring)	2	1
Diaphragm	3	1
None	26	9
Other	16	6

### Awareness and knowledge of the female condom prior to study

Most women (71% (202/284)) indicated that they were aware of the female condom prior to participating in the study. Of these, many reported that this was via the internet (42% (84/202)) or school (27% (54/202)), although one-fifth could not recall how they had become aware (21% (43/202)). Less than 10% (19/202) reported learning about the female condom from a health care provider.

The majority of participants knew that the female condom can be used to prevent pregnancy and STIs (68% (193/284)) and can be used in combination with other methods of contraception (65% (185/284)). Fewer were aware that it can be purchased without a prescription in a pharmacy (42% (120/284)) or online (44% (124/284), or that it is 95% effective as a contraceptive when used correctly with every episode of intercourse (34% (96/284)). The survey did not include a question regarding effectiveness during ‘typical’ use.

### Experiences and views of the female condom during study

The majority of women (79% (223/284)) reported using at least one of the female condoms provided during sex (1 used = 55% (156/284); 2 used = 14% (39/284); 3 used = 10% (28/284)). The remaining participants either attempted to insert the female condom (11% (30/284)) or did not progress past opening the packet (11% (31/284)). Less than half saw a demonstration of how to use the female condom before attempting use, either by video (44% (124/284)) or with a clinician in person (2% (6/284)). Participants also primarily inserted the female condom by themselves (77% (158/205)), rather than with the assistance of their sexual partner.

As shown in Table 3, 51% (104/205) of participants experienced some level of difficulty when inserting the female condom. However, removal of the female condom was reported to be relatively easy (81% (167/205)).

**Table 3:** Ease of inserting and removing the female condom (*n* = 205*).

		Insertion		Removal	
	Rating	*n*	%	*n*	%
Very difficult	1	31	15	5	2
**↑**	2	73	36	13	6
Neither difficult/easy	3	44	21	20	10
**↓**	4	34	17	41	20
Very easy	5	23	11	126	61

* Total number of participants (n = 205) comprises women who reported using or attempting to use at least one female condom (n = 253), excluding those who did not provide an answer to these questions (n = 48).

Participants were asked to compare the ‘feeling of sex’ with a female condom in comparison to without a condom and with a male condom. Most rated the female condom as comparatively worse than when using no condom (81% (160/197)) or the male condom (60% (122/204)); see Table 4). However, approximately half (51% (105/204)) of participants rated the sensation and comfort of the female condom during sex as the same or better than the male condom, and two-thirds (66% (137/204)) that it provided the same or better lubrication (see Table 5).

**Table 4:** Comparison of the female condom to no condom and the male condom*.

		No condom (n = 197)		Male condom (n = 204)	
	Rating	*n*	%	*n*	%
A lot worse	1	85	43	45	22
↑	2	75	38	77	38
No different	3	24	12	39	19
↓	4	10	5	31	15
A lot better	5	3	2	12	6

* Total number of participants comprises women who reported using or attempting to use at least one female condom (n = 253), excluding those who reported that they had not had sex without a condom or with a male condom.

**Table 5:** Comparison of the female condom to the male condom (*n* = 204).

	Sensation		Comfort		Spontaneity		Lubrication	
	*n*	%	*n*	%	*n*	%	*n*	%
Better	33	16	28	14	15	7	44	22
The same	72	35	71	35	54	27	93	46
Worse	99	49	105	51	135	66	67	33

Participants were also invited to indicate whether they found the female condom helpful for being in control of their own STI protection and/or contraception; approximately half agreed (52% (105/204)), while fewer disagreed (28% (57/204)) or were unsure (21% (42/204)).

When asked whether they would consider using the female condom again, responses in relation to using it for contraception were evenly distributed between yes (40% (103/260)) and no (41% (106/260)). More participants reported they would consider using it again for STI prevention (51% (133/260)). Less than half of the sample would consider recommending the female condom to others (43% (112/260)) (Table 6). The price that women reported being willing to pay for one female condom was typically between $1–2 (67% (174/260)) or $3–5 (29% (76/260)), with a small proportion willing to pay $5 or more (4% (10/260)).

**Table 6:** Consider future use of the female condom and recommending to others (*n* = 260).

	Yes		No		Unsure	
	*n*	%	*n*	%	*n*	%
Consider using again for contraception	103	40	106	41	51	20
Consider using again to prevent STIs	133	51	85	33	42	16
Would recommend to others to use	112	43	73	28	75	29

* 24 women did not respond to these questions.

## Discussion

Most participants were aware of the female condom before participating in the study; however, practical experience of using this method was limited. Previous awareness of the female condom was mainly via the internet, and to a lesser extent school. Few women had learned about the female condom from a health care provider. Most knew that the female condom could prevent both pregnancy and STIs, and could be used with other methods of contraception, however many were unaware of its effectiveness as a contraceptive when used correctly with every episode of intercourse or that they could purchase female condoms online or in a pharmacy without a prescription. While women’s insertion experiences were varied in the study, approximately half rated the female condom as comparable to male condoms across several factors, including sensation and comfort, and two-thirds better in terms of lubrication. Between forty to fifty percent of women would also consider recommending the female condom to others or using it again themselves in future, particularly for STI prevention.

While most studies exploring the experiences of women using the female condom have been conducted with specific communities or in low-income countries [8,17,25], this is the first study to examine the experiences of women in Australia. Findings may inform health promotion efforts, clinical consultations and sexual health counselling guidelines.

In the current study, many women reported experiencing some difficulty in inserting the female condom. Previous research has similarly reported initial insertion difficulties [e.g., 26], however others have shown that these early challenges become easier with continued use [e.g., 17,25,27,28]. For example, one study with South African women with a history of engaging in unprotected sex found that after 2–3 attempts, women were no longer experiencing insertion-associated difficulties [25]. More generally, these difficulties have been attributed to the ‘newness’ of the method, and therefore women’s unfamiliarity with the female condom [17,29]. Given that the majority of our sample only used one of the three female condoms provided, and did not have previous experience using the method nor saw a demonstration prior to use, participants’ experiences of inserting are likely to improve as they become more familiar with the method [25,30]. This could be facilitated by watching a demonstration online [31,32] or with the advice and support of a health professional [33,34].

To support women’s use of the female condom, contraceptive and STI consultations could include a discussion of the challenges that may arise when first using the female condom [34] and the provision of reliable educational resources [35]. Practicing inserting the female condom before using with a sexual partner may also be beneficial [33]. Health professionals could also demonstrate insertion of the female condom with visual aids or demonstration models [e.g., 33,34]. Similar to other studies [35], however, our results suggest that health professionals are not the main or only source of information about the female condom. Therefore, a multidimensional approach to education would be of value, including the participation of pharmacists, parents and teachers, as well as educational tools that may be accessed online.

Most women in this study preferred the feeling of sex without a condom to sex with a female condom, which is a common finding among previous studies of both the female condom [e.g., 17,36–39] and male condom [e.g., 40–42]. A more useful comparison, therefore, may be across two barrier forms of protection; male condoms and female condoms. Approximately half of our sample reported that the female condom was comparable to the male condom in terms of sensation and comfort. Other acceptability studies have reported similarly mixed feelings in terms of their preferences between female and male condoms for both women [63%; 43] and men [44%; 44]. Participants in the current study also reported the female condom as having the same or better lubrication than the male condom. This finding supports that of a study among South African women, where 63% of the sample reported that the amount of lubricant was ‘just right’ [45]. However, the female condom was viewed by women in this study as allowing for less spontaneity than the male condom. In contrast to this finding, in other studies female condoms have been associated with greater sexual spontaneity as they can be inserted prior to sex [21,23]. This is a feature of the female condom which could be emphasised during relevant clinical consultations and health promotion activities.

When considering future use of the female condom, participants’ responses showed a preference for use for STI prevention rather than for use as contraception. Approximately half of women also viewed the female condom as helpful for being in control of their own STI protection and/or contraception. As there is already a wide range of effective female-initiated contraception methods available, the female condom may be particularly useful for women seeking control of their own STI prevention. In a study undertaken with low-income Puerto Rican woman at-risk of HIV/AIDs, reasons for trying the female condom were primarily associated with STI prevention (63%) rather than contraception (5%) [29]. Findings from another study with women and couples living in the United States suggested that those who used hormonal contraception were more likely to pair this with the female condom for STI prevention [46]. Supporting women to be in control of their own STI protection if desired may also negate the need to negotiate male condom use, particularly for young women [46] or those who are in power-imbalanced relationships [47–49].

Although a proportion of women in the study reported they would consider using the female condom again or recommend it to others, there are accessibility barriers to consider. Female condoms are often reported as being difficult to find and/or expensive to buy, both in Australia and globally [7,48,50–57]. Anecdotally, an Australian news editorial in 2019 described the limited stores that stocked female condoms, as well as their high cost [58]; despite the FC2 that is currently on the market costing 30% less than the original design (FC1) [59]. According to the websites of Australian online recommended retailers, an FC2 three pack costs between $10–15 [60–62]. Factors contributing to the relatively high cost of female condoms include low consumer demand likely due to their limited availability as well as consumer familiarity and acceptance of the product [63]. Only a small proportion of participants in our study had used the female condom previously, and, on average, reported that they would be willing to pay $1–2 per female condom. Increasing the demand for female condoms may reduce costs and other access issues for those who wish to use this method. For example, utilising health professionals to raise awareness, as well as inclusion in school-based sexuality education and contraception/STI health promotion activities, may contribute to increasing demand for female condoms.

### Limitations and future research

This is the first study in Australia to report on the views and experiences of women in relation to their use of the female condom. We were able to provide each participant with three female condoms, rather than only recruiting those who had used them in the past or were in a position to purchase them for the study. This may have resulted in a greater sample of diverse women. Although the majority of the sample was young (<35 years), they appeared to be otherwise representative of the general population in many aspects, including Aboriginal background, remoteness area and contraception use. However, several limitations of the sample should also be considered. Most women reported being born in Australia, so the views of those born in other countries were less represented. The study was also conducted in English only. Most women were in a committed relationship, and therefore may be less representative of women in other types of relationship or in no relationship. Finally, as this was a small exploratory study, we limited the eligibility criteria to ‘females who are heterosexually active’. We recognise this is very targeted and may exclude particular people, including men, trans and gender diverse people, and others in the LGBTIQ+ community.

This study provides a foundation for future research on the female condom in Australia. While we have explored the experiences of women, the perspectives of their sexual partner(s) are likely to be an important factor in terms of acceptability and uptake, which warrants further investigation. Similar studies could also be considered to ensure representation from a range of perspectives, including trans and gender diverse people, and others in the LGBTIQ+ community. Longer-term studies would also be useful in determining if any initial difficulties or concerns about the female condom may be overcome with continued use.

### Conclusion

The current study provides valuable insights regarding women’s views and experiences of using the female condom. Although many women experienced some difficulty in using the female condom, many would consider using it again in future or recommending it to others, particularly as a method of STI prevention. These findings highlight the importance of having a female-initiated method for STI prevention, and suggest the need for further clinical, education and health promotion efforts regarding correct use of the female condom. To reduce accessibility barriers, it will also be important to address the cost and availability of the female condom in the Australian market. Future research should explore the views and experiences of others, including men, trans and gender diverse people and others in the LGBTIQ+ community.
